# Mistreatment with maggot therapy in diabetic foot ulcer causing an amputation

**DOI:** 10.1002/ccr3.8041

**Published:** 2023-10-10

**Authors:** Arefeh Babazadeh, Soheil Ebrahimpour, Zeinab Mohseni Afshar, Sara Mohammadnia

**Affiliations:** ^1^ Infectious Diseases and Tropical Medicine Research Center, Health Research Institute Babol University of Medical Sciences Babol Iran; ^2^ Kermanshah University of Medical Sciences Kermanshah Iran; ^3^ Student Committee Research Babol University of Medical Sciences Babol Iran

**Keywords:** diabetes mellitus, diabetic foot ulcer, maggot therapy, mistreatment

## Abstract

Maggot therapy is one of the treatments used in diabetic foot ulcer management. But if we do not pay attention to the indications and contraindications of it, there might be a failure in the treatment.

## 1 CASE REPORT

A 52‐year‐old man with a 14‐year history of Type 2 diabetic mellitus was admitted to the hospital with fever and diabetic foot ulcer (DFU) on three sites of both of his feet in June 2022. He had no other comorbidities. To control his blood sugar level, he had undergone a pharmacological treatment with sitagliptin/metformin tablet (50/1000 mg, BD) and glibenclamide (5 mg, BD). He smoked 30 packs of cigarettes a year. He said that the wounds were formed about 2 weeks ago and he took care of them with homemade herbal therapies, but the wounds got worse.

When his fasting blood sugar was checked in the hospital, it was at 192 mg/dL and his HbA1c was at 7% so a consultant by an endocrinologist was asked. On the day of admission, his C‐reactive protein was at 182 mg/L. His WBC was at 17200 units/mm^3^ with 85% neutrophil. Ceftriaxone injection (1 g, TDS) and Clindamycin injection (300 Mg, TDS) were started for him at the time. Then, orthopedists cleaned the wound and debrided the necrosed tissues. A color Doppler ultrasonography in the evaluation of the DFU did not show any abnormality. On the second and third day of admission, and again on the sixth day of admission, he had fevers that were controlled by intravenous paracetamol injections (1 g). On the third day of admission, vancomycin injection (750 mg, BD) and piperacillin (4.5 g, TDS) were started. On the fourth day surgery was performed to debride the necrotic parts of the ulcer. The patient planned to leave the hospital on the seventh day of admission and stopped following our treatments.

**FIGURE 1:**
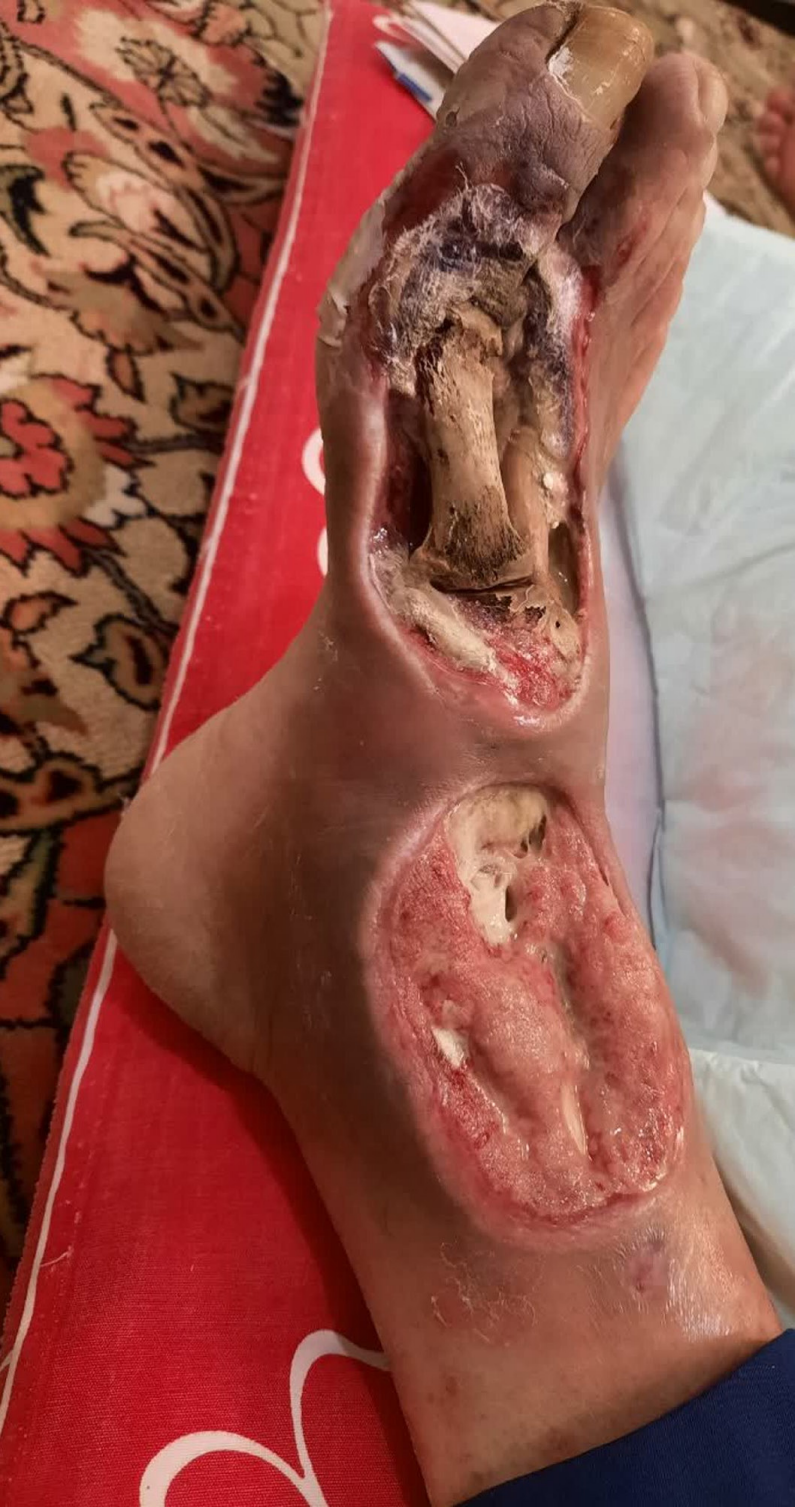
Diabetic foot ulcer after maggot therapy.

**FIGURE 2:**
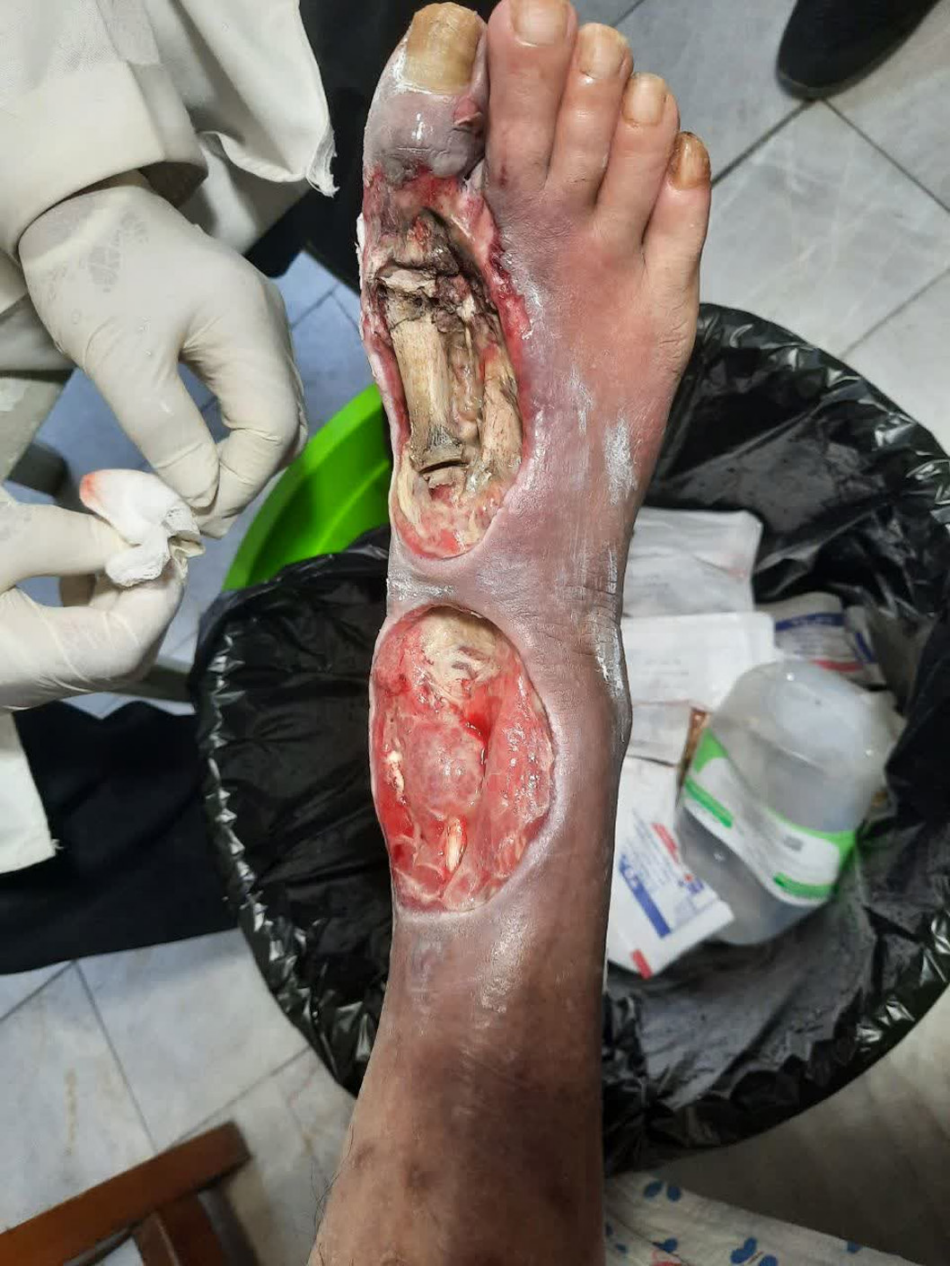
Diabetic foot ulcer after maggot therapy.

**FIGURE 3:**
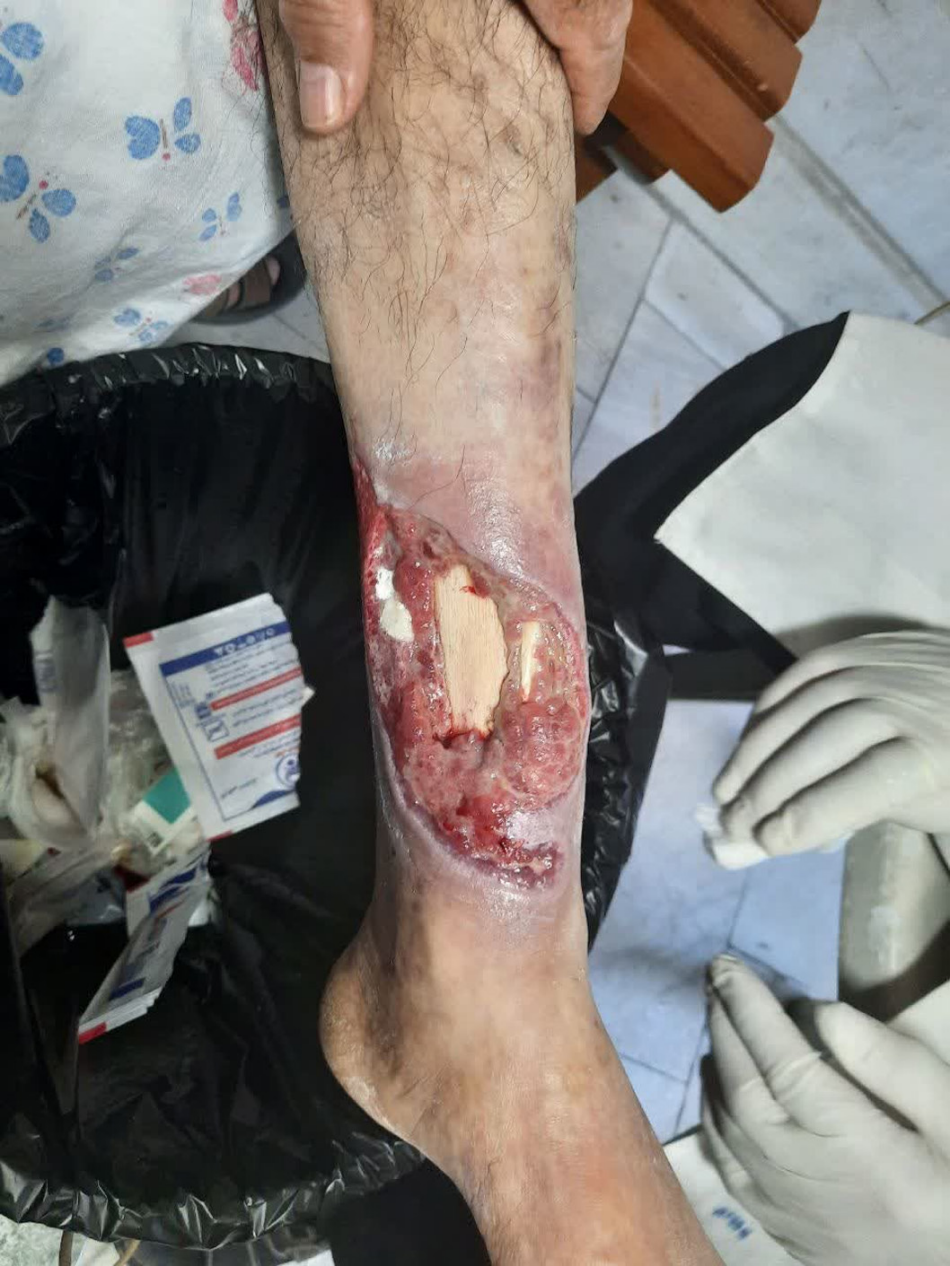
Diabetic foot ulcer after maggot therapy.

He did not continue the treatments in any other health clinics, instead, he attended a fungal specialist. The specialist recommended maggot therapy. However, the ulcer worsened after just one session of therapy and the larva ate all the granulation tissue and healthy normal tissue until the bones were seen (Figure 1, 2, 3). He was admitted to the hospital again 2 months after his last admission and below‐knee amputation was performed on his right foot.

## 2 DISCUSSION

Maggot therapy is believed to be an effective treatment as a debridement therapy in chronic wounds such as DFU all around the world.^1^

However, there are some contraindications of maggot therapy such as very dry wounds, open wounds in the abdominal cavity, pyoderma gangrenosum in patients with immunosuppressive therapy, and septic, arthritis wounds heavily contaminated with pseudomonas aeruginosa.^2^, ^3^

In this case, pseudomonas aeruginosa infection and tendon exposure are the reasons that maggot therapy must be avoided.

## AUTHOR CONTRIBUTIONS

**Arefeh Babazadeh:** Writing – original draft; writing – review and editing. **Soheil Ebrahimpour:** Writing – review and editing. **Zeinab Mohseni Afshar:** Writing – review and editing. **Sara Mohammadnia:** Writing – original draft; writing – review and editing.

## CONFLICT OF INTEREST STATEMENT

The authors have no conflict of interest to declare.

## CONSENT

Written informed consent was obtained from the patient to publish the current case report.

## Data Availability

The data that support the findings of this study are available on request from the corresponding author. The data not publicly available due to privacy or ethical restrictions.
